# Development of Path Generation and Algorithm for Autonomous Combine Harvester Using Dual GPS Antenna

**DOI:** 10.3390/s23104944

**Published:** 2023-05-21

**Authors:** Kyuho Lee, Hyohyuk Choi, Junghun Kim

**Affiliations:** Autonomous Vehicle Intelligent Robotics, Industrial Convergence Interdepartmental Program, Hongik University, Seoul 04066, Republic of Korea; rbgh0214@mail.hongik.ac.kr (K.L.); gyehgur123@mail.hongik.ac.kr (H.C.)

**Keywords:** path generation, path tracking, autonomous combine harvester, dual GPS antenna

## Abstract

Research on autonomous driving technology is actively underway to solve the facing problems in the agricultural field. Combine harvesters used in East Asian countries, including Korea, are tracked-type vehicles. The steering control system of the tracked vehicle has different characteristics from the wheeled vehicle used in the agricultural tractor. In this paper, a dual GPS antenna-based autonomous driving system and path tracking algorithm were developed for a robot combine harvester. An α-turn-type work path generation algorithm and a path tracking algorithm were developed. The developed system and algorithm were verified through experiments using actual combine harvesters. The experiment consisted of an experiment with harvesting work and an experiment without harvesting work. In the experiment without harvesting work, an error of 0.052 m occurred during working driving and 0.207 m during turning driving. In the experiment where the harvesting work was carried out, an error of 0.038 m occurred during work driving and 0.195 m during turning driving. As a result of comparing the non-work area and driving time to the results of manual driving, the self-driving experiment with harvesting work showed an efficiency of 76.7%.

## 1. Introduction

Recently, the FAO food price index has hit an all-time high, so grains show high inflation. As a result, food security is becoming an important issue as food supply and demand can become unstable in countries that rely on grain imports [1]. However, in most developed countries, agriculture is less attractive among the younger generation because its wages are less than typical white-collar jobs, and the work is more complicated than typical white-collar jobs. Therefore, the agricultural population is gradually aging and decreasing, and food security is becoming increasingly dangerous due to decreased productivity [2]. In order to solve these problems, interest in ‘precision agriculture’ that maximizes crop production while minimizing input resources by using autonomous driving technology and ICT (Information and Communication Technology) is increasing [3].

In precision agriculture, autonomous driving technology has many advantages, such as replacing labor, increasing work efficiency, optimizing work paths, and saving fuel. Research on autonomous driving of agricultural machinery actively began in the 1990s [4,5,6]. In particular, since 2000, the United States government has provided a GPS (Global Positioning System) that has not intentionally added noise to the private sector, and as the precision of the GPS has increased, research on autonomous driving using the GPS has increased as well [7,8]. In addition, research on autonomous driving using gyro sensors, cameras, or laser sensors has also been steadily conducted [9,10,11,12].

Agriculture has various processes, such as crop cultivation, harvesting and processing, and distribution. Much labor is required for the harvest process, and at least two workers transport the harvested crop. In addition, the combine harvester used for harvesting operations is essential because timely harvesting significantly impacts the harvest’s product value. It is challenging to apply the autonomous driving combine harvester research. In most East Asian countries, including Korea, the combine harvester is the tracked-type vehicle. In the case of a vehicle having a wheel-type steering device, such as a tractor, the driving direction is controlled by controlling the steering angle of the wheel, whereas in the case of a tracked vehicle, the speed of the left and right tracks is controlled to control the driving direction. Therefore, applying the autonomous driving algorithm studied for a vehicle with a wheel-type steering system to a tracked vehicle is not appropriate. 

An appropriate path-planning algorithm must be considered for efficient harvesting work using the autonomous combine harvester. In the case of Korea, it has the characteristics of a relatively small field size and conducts harvesting work in the headland. More precise path generation and search technology are needed [13]. Accordingly, researchers in Korea, China, and Japan have conducted research and development algorithms for autonomous combine harvesters and verified them using computer simulation methods [14,15,16,17,18,19,20,21]. Most of the existing papers have been on self-driving using GPS and gyroscopes. However, as the performance of computers has improved recently, research on autonomous driving using cameras is continuously being conducted [22,23,24]. In addition, studies were conducted to distinguish the working area from the non-working area using LiDAR [25,26].

In this paper, on the premise of mass production, we propose an autonomous driving system and driving algorithm for an autonomous combine harvester and a path generation algorithm suitable for the characteristics of rice fields in Korea. Since the research was conducted on the premise of mass production, a low-cost dual-antenna GPS was used instead of the expensive LiDAR sensor, IMU sensor, or GPS that had been studied previously. For data transmission and reception with the existing combine harvester, the communication system used CAN 2.0B standard. In addition, to verify algorithms, experiments were conducted while harvesting in Korean rice fields, not in simulations.

## 2. Materials and Methods

### 2.1. Combine Harvester Hardware System

**Figure 1:**
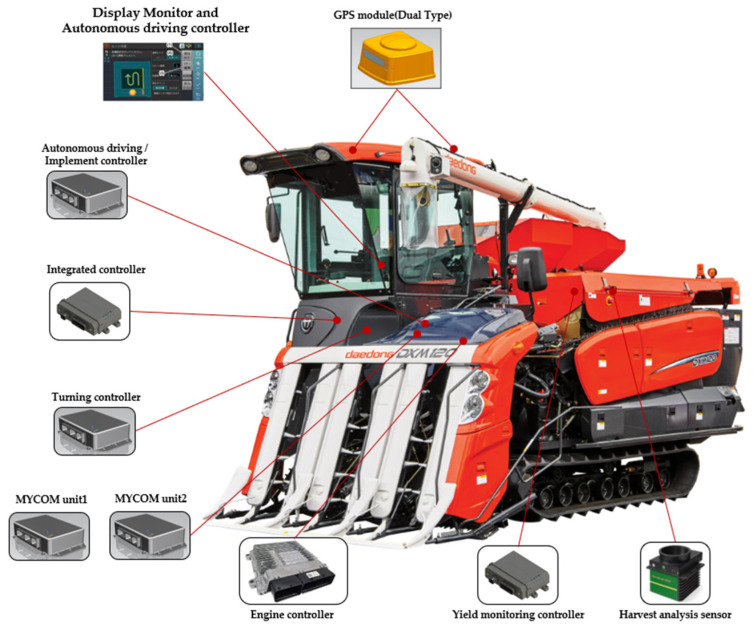
Combine harvester hardware system mounting location.

**Figure 2:**
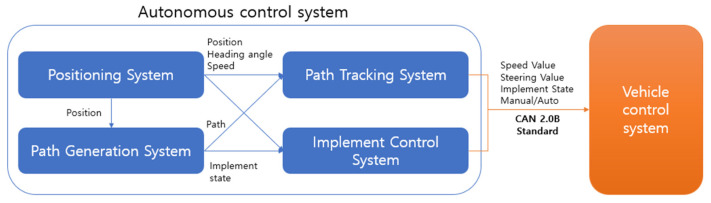
Combine harvester autonomous system structure diagram.

In this paper, a six-row combine harvester was used, and the overall length of the combine harvester was 4980 mm, the overall width was 2430 mm, and the total height was 2780 mm. The control system of the combine harvester was divided mainly into an autonomous driving control system and a vehicle control system. The autonomous control system comprises a positioning system, a path generation system, a path tracking system, and an implementation control system. The vehicle control system has two operation statuses, autonomous and non-autonomous driving. The non-autonomous driving function can be manually controlled similar to an existing combine harvester. In the autonomous driving function, the vehicle control system executes the command value received from the autonomous driving control system. Data transmission and reception between the two control systems are communicated using the CAN 2.0B standard. The hardware system of the combine harvester is controlled by dividing it into seven parts according to the control function. The control function is divided into reaping, threshing, discharging, driving speed, steering, engine RPM, autonomous driving/work, display, and operation control. Figure 1 shows the mounting location of the combine harvester hardware system. Figure 2 shows the structure of the combine harvester system and the data moving between each system.

### 2.2. Combine Harvester Autonomous System

The autonomous control system of the combine harvester was configured using the LabView program on an industrial PC.

#### 2.2.1. Positioning System

The positioning system uses GPS sensors to obtain the current position, heading angle, and speed information of the combine harvester and provides them to the path generation and path tracking systems.

**Figure 3:**
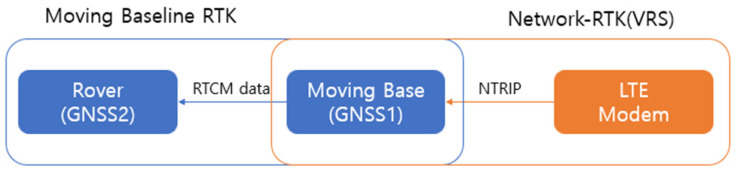
Positioning System Diagram.

When the GPS sensor is used without additional calibration, the error measures the position with an accuracy of several centimeters to several tens of centimeters. However, a more precise position is required for harvesting. To compensate for these shortcomings, Network-RTK (a Real Time Kinematic (VRS-GPS) method) was used to create a virtual reference system (VRS) near the mobile station using data received from the permanent monitoring station and to calculate system errors and transmit correction values to the mobile station [27,28]. However, if only one GPS sensor is used, it is impossible to check the current direction of the combine in a stopped state. In order to compensate for these disadvantages, the moving baseline RTK method was used using two GPS sensors. Moving baseline RTK sets up two GPS receivers as the base and rover. The base and rover can obtain a carrier phase from each satellite, and the base converts measurements into RTCM(Radio Technical Commission for Maritime Services)-type correction information and transmits it to the rover [14]. The position information of the combine harvester was used by converting the longitude and latitude coordinates received from the RTK-GPS into UTM52N coordinates, a flat rectangular coordinate system. The RTK-GPS sensor is u-blox ZED-F9P™, and the specifications are shown in Table 1. Figure 3 is a simplified diagram of the positioning system.

In the case of latitude and longitude values received from the GPS, they are angular values. They cannot be calculated as distance and direction angles required for autonomous driving of the combine. Therefore, the latitude and longitude of the wgs84 coordinate system received from the GPS were converted into the UTM coordinate system, which is a two-dimensional projected coordinate system, and then used.

**Figure 4:**
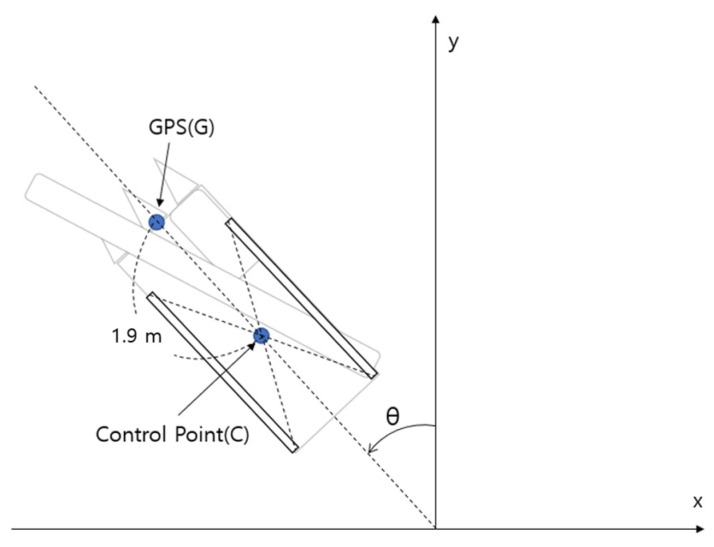
Translation of GPS coordinates.

Since there is a gap between the combine harvester position coordinates received from the GPS and the control point of the combine harvester, the coordinate value fluctuates often when the combine harvester turns, which needs to be corrected. Therefore, as shown in Equation (1), the GPS coordinates were moved in parallel to the control point using the direction angle of the combine and separation distance of 1.9 m. Figure 4 shows the difference between the GPS mounting location and the center of the control point of the combine harvester.

(1) $$\left[\begin{array}{c} x_{c}\\ y_{c} \end{array}\right]=\left[\begin{array}{c} x_{g}\\ y_{g} \end{array}\right]-\left[\begin{array}{c} L\sin\theta \\ L\cos\theta \end{array}\right]$$

#### 2.2.2. Path Generation System

The path generation system generates work path data through coordinate data provided by the positioning system. Work path data is expressed as a RDDF (Route Data Definition File), comprising six columns. The index indicates the number of waypoints. Latitude is the latitude value converted from WGS84 coordinates to UTM-52N coordinates. Longitude is the longitude value converted from WGS84 coordinates to UTM-52N coordinates. The speed indicates the forward and backward speed of the combine harvester. A positive number means forward, and a negative number means backward. The status of the combine harvester in the work path indicates whether the combine harvester is in the work area or the turning area. Implement status indicates the status of the implement, one indicates that the implement is working by lowering the implement, and zero indicates that the implement is not operating by raising the implement. Table 2 shows the composition of the RDDF.

#### 2.2.3. Path Tracking System

The path tracking system is a system that controls the combine harvester using the current position, heading angle, and speed of the combine from the positioning system. Based on the RDDF received from the path generation system, the system controls the combine harvester’s speed and steering. The combine harvester’s forward, backward, and steering values are calculated using CAN communication and are transmitted to the vehicle control system.

#### 2.2.4. Implement Control System

The implement control system is a system that controls the operation of the implement based on the positioning of the current combine harvester received from the RDDF and the positioning system received from the path generation system. The value of the operating machine is transmitted to the vehicle control system using CAN communication.

### 2.3. Combine Harvester Path Generation

**Figure 5:**
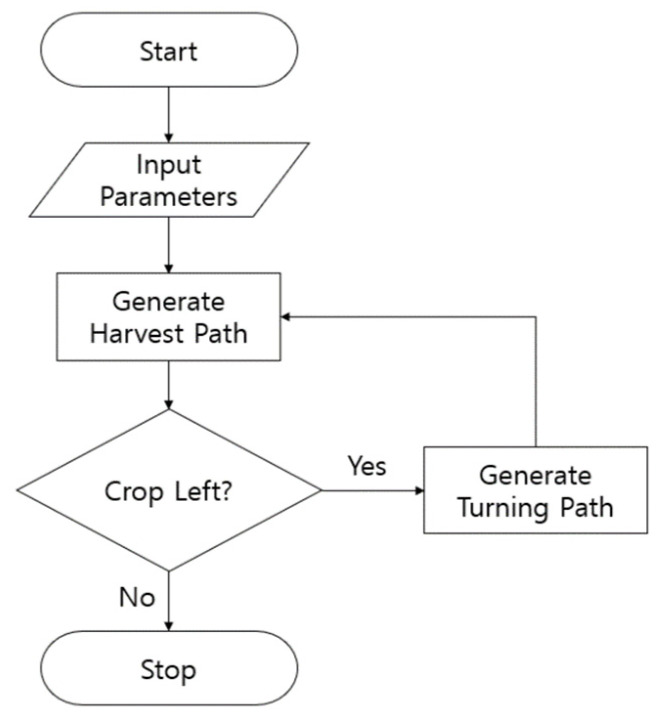
Flowchart of path generation algorithms.

The path generation algorithm consists of the parameter input, harvesting path, and turning path generation. The harvesting path was generated in the form of a straight path, and the turning path was generated by a switchback turning method. Figure 5 shows the flow of the path generation algorithm.

#### 2.3.1. Parameter Input

Parameters required for path generation include the work areas, combine harvester, and turning types. Four vertex coordinate inputs of the working rice paddy are required for the parameter of the working area. The four vertex coordinates are acquired when performing manual work to generate the space necessary for turning.

The parameters of the combine harvester include the width of the working implement, the harvesting speed, the turning speed, and the distance between the front of the working implement and the control point. When controlling the working implement, the working implement command is issued based on the control point. For this reason, the distance between the working implement and the control point was entered to correct the timing of the descent of the working implement during the harvesting operation.

Parameters for the working path include waypoint spacing, look-ahead distance, the turning radius, and over-run distance to prevent damage to the unworked crop area during turning. The waypoint interval was set to 0.2 m. Table 3 shows the parameters required for path generation.

#### 2.3.2. Harvesting Path Generation

In small rice paddies, such as in Korea and Japan, a harvest method cut inward from the edge of the rice paddy is widely used. The reason for the counter-clockwise harvesting is that when discharging after the harvesting, the harvesting vehicle must be on the right side of the combine harvester to make moving the auger onto the harvesting vehicle easier.

**Figure 6:**
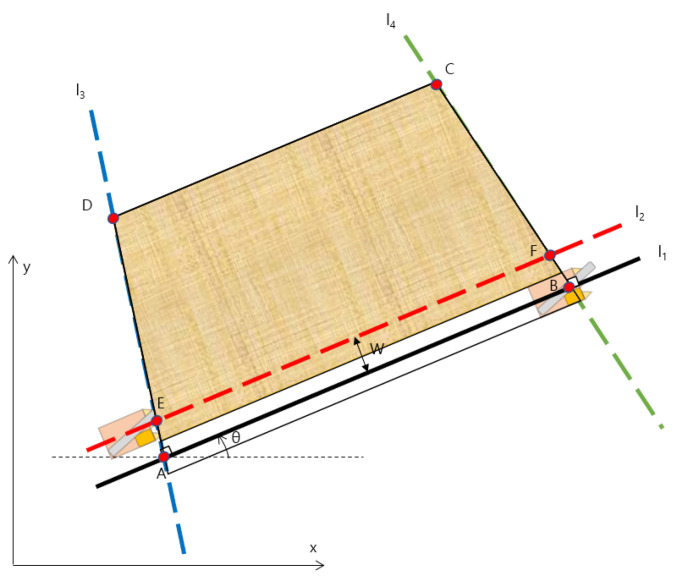
Description of how to move reference points.

The harvesting path was generated by linearly interpolating the two reference points at waypoint intervals. When the generation of the corresponding harvesting path is completed, the reference point is moved to the following harvesting path. The reference point movement method is shown in Figure 6. Based on the straight line L2 moving in parallel from straight line L1 to the next harvesting work area by the width of the working machine, the intersection point with L3 is set as the next reference point E, and the intersection point with straight line L4 is set as the next reference point F. The harvesting path is continuously generated by the above method, and when the distance between the straight line L1 and point C is less than the width of the work implement, the path generation is terminated.

#### 2.3.3. Turning Path Generation

A turning operation is required for the combine harvester to go to the following harvesting path after harvesting. The turning method is the α-turn method, and the generation of the turning path was created as a Dubin’s Path consisting of straight lines and arcs considering the limitations of damage to unharvested crops and to avoid crossing the rice paddy banks.

The α-turn turning method is also called the switchback turning method and turns to the following harvest work path using straight and backward motions. The combine harvester is a tracked vehicle, so it is possible to turn it in place. Since the soil is soft due to the characteristics of irrigation agriculture in the rice paddy, it is possible to damage the soil on the track of the combine harvester, so turning it in place is avoided.

**Figure 7:**
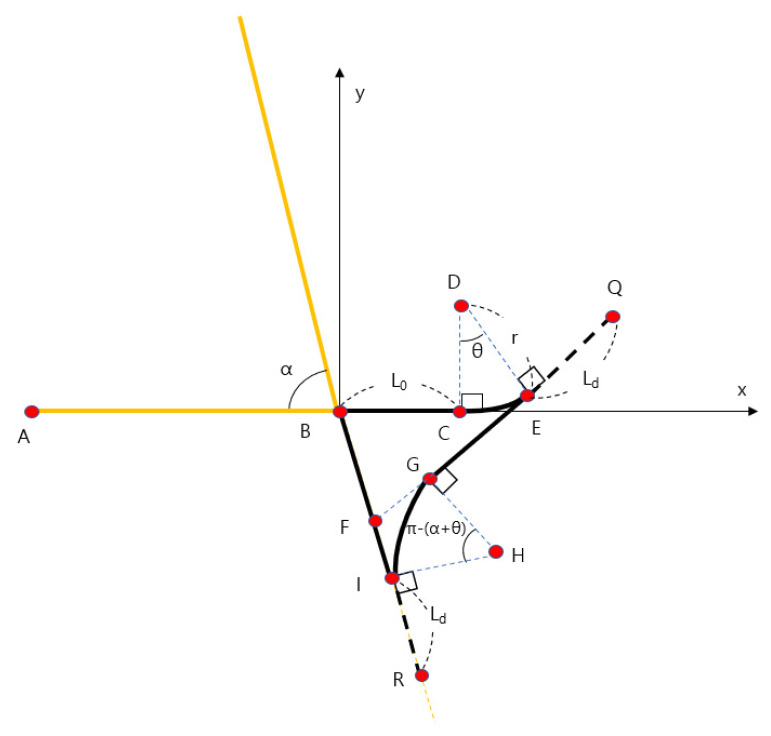
α-turn Turning Path Reference Point.

As shown in Figure 7, the α-turn turning path consists of a path starting at point B and going straight by the over-run distance L0, a path that rotates along an arc of a turning radius r and a turning angle θ. The turning path was generated by dividing the length of the arc by waypoint intervals, dividing the angles into equal parts, and then rotating and converting the starting point of the turning path by the angle divided into equal parts based on the turning center. The straight path was generated by linear interpolating as much as the waypoint interval. The coordinate value of each reference point is shown in Equations (2)–(10).

(2) $$x_{c}=L_{0},\;y_{c}=0$$

(3) $$x_{d}=L_{0},\;y_{d}=r$$

(4) $$x_{e}=L_{0}+r\sin\theta ,\;y_{e}=r\left(1-\cos\theta \right)$$

(5) $$x_{q}=L_{0}+r\sin\theta +L_{d}\cos\theta ,\;y_{q}=r\left(1-\cos\theta \right)+L_{d}\sin\theta$$

(6) $$x_{f}=\frac{\tan\theta \left(L_{0}+r\sin\theta \right)-r\left(1-\cos\theta \right)+L_{d}\sin\theta }{\tan\theta +\tan\alpha }\;,\;y_{f}=-\tan\alpha \frac{\tan\theta \left(L_{0}+r\sin\theta \right)-r\left(1-\cos\theta \right)}{\tan\theta +\tan\alpha }$$

(7) $$x_{g}=x_{f}+r\tan\frac{\pi -\left(\theta +\alpha \right)}{2}\cos\theta \;,\;y_{g}=y_{f}+r\tan\frac{\pi -\left(\theta +\alpha \right)}{2}\sin\theta$$

(8) $$x_{i}=x_{f}+r\tan\frac{\pi -\left(\theta +\alpha \right)}{2}\cos\alpha \;,\;y_{i}=y_{f}-r\tan\frac{\pi -\left(\theta +\alpha \right)}{2}\sin\alpha$$

(9) $$x_{h}=x_{i}+r\cos\left(\frac{\pi }{2}-\alpha \right)\;,\;y_{h}=y_{i}+r\sin\left(\frac{\pi }{2}-\alpha \right)$$

(10) $$x_{r}=x_{i}+L_{d}\cos\alpha ,\;y_{r}=y_{i}-L_{d}\sin\alpha$$

$L_{0}$: Over-run distance.

$L_{d}$: Look-ahead distance.

r: Turning radius of combine harvester.

θ: Turning angle.

α: Corner angle of work area.

### 2.4. Combine Harvester Control

#### 2.4.1. Lateral Control

The combine harvester’s lateral control was implemented by referring to the kinematic elements of the combine harvester, the skid steering method, and the pure pursuit algorithm, which is an existing path-tracking algorithm.

In order to consider the kinematics of the combine, we assumed that the combine harvester is located on the inertial coordinate system’s horizontal plane (X_o_, Y_o_, Z_o_). The reference coordinate axis (X_p_, Y_p_, Z_p_) of the combine harvester is indicated based on the center of gravity of the combine harvester. The center of gravity of the combine harvester is displayed as in the inertial coordinate system (x, y, z). In an actual combine harvester, shaft displacement Z_p_ occurs while harvesting but this displacement is minimal compared to the displacement on a plane (X_p_, Y_p_), as such, the shaft displacement is not considered.

**Figure 8:**
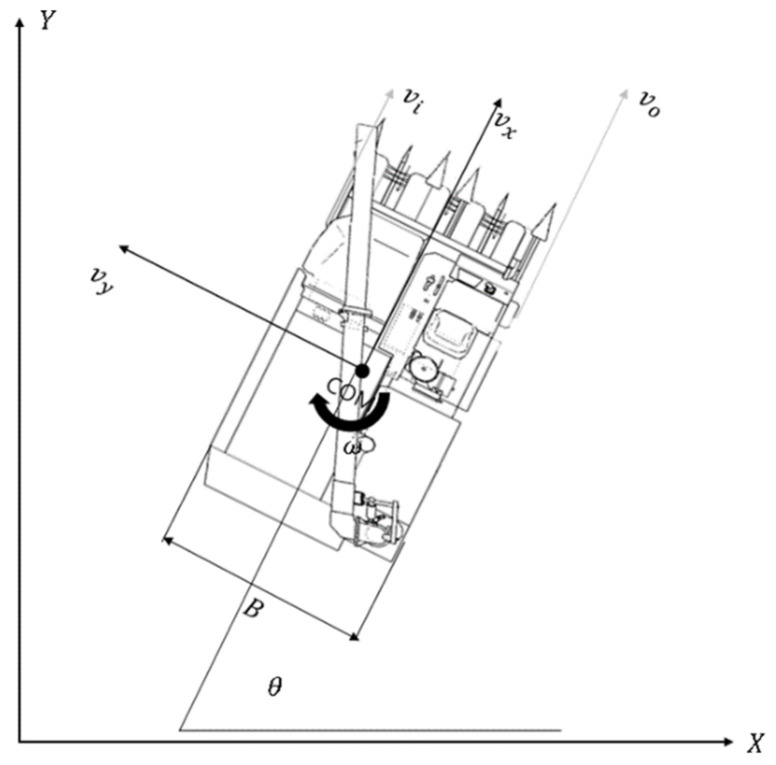
Freebody diagram of combine harvester.

The combine harvester moves only in the (X_p_, Y_p_) plane, so we expressed velocity as $v_{x},\;v_{y}$. The angular velocity of the combine is denoted as $\vec{v}={\left[v_{x},\;v_{y},\;0\right]}^{T}$ because there is no displacement in the (X_p_, Y_p_) plane. Assuming that the center of gravity vector of the combine harvester is $\vec{p}={\left[x,\;y,\;\theta \right]}^{T}$, the speed vector of the combine harvester is expressed as $\vec{v}={\left[\dot{x},\;\dot{y},\;\dot{\theta }\right]}^{T}$. Figure 8 shows the free-body diagram of the combine harvester.

Skid steering is a method of steering by using the difference between the speed of the inner and outer crawler track of the combine harvester. By knowing the width of the combine harvester and calculating the turning radius distance in a given path, we can obtain the speed ratio of the inner and outer crawler tracks, and the formula is defined as 12. When the combine harvester runs, lateral slip occurs between the ground and the combine, but this paper does not consider this.

(11) $$v_{o}:v_{i}=R+\frac{B}{2}:R-\frac{B}{2}$$

(12) $$\frac{v_{o}}{v_{i}}=\frac{R-\frac{B}{2}}{R+\frac{B}{2}}=i$$

(13) $$R=\frac{B\left(1+i\right)}{2\left(1-i\right)}$$

$v_{o}$: Speed of combine harvester’s outer crawler track.

$v_{i}$: Speed of combine harvester’s inner crawler track.

R: Turning radius of combine harvester.

B: Width of combine harvester.

i: Speed ratio of combine harvester’s crawler tracks.

Pure pursuit is one of the most representative algorithms, along with the Stanley Method as one of the autonomous driving path tracking algorithms. Draw an arc using the vehicle’s current coordinate point and target point and calculate the steering angle through the diameter of the arc. The distance from the current coordinate point of the vehicle to the target point is the ‘Look-Ahead Distance’ (LAD). When the LAD changes, the diameter of the arc changes as the target point changes, and the steering angle also changes accordingly. In the pure pursuit algorithm, if the LAD is small, the performance of tracking the path is improved but oscillation occurs due to the large steering angle change of the vehicle.

**Figure 9:**
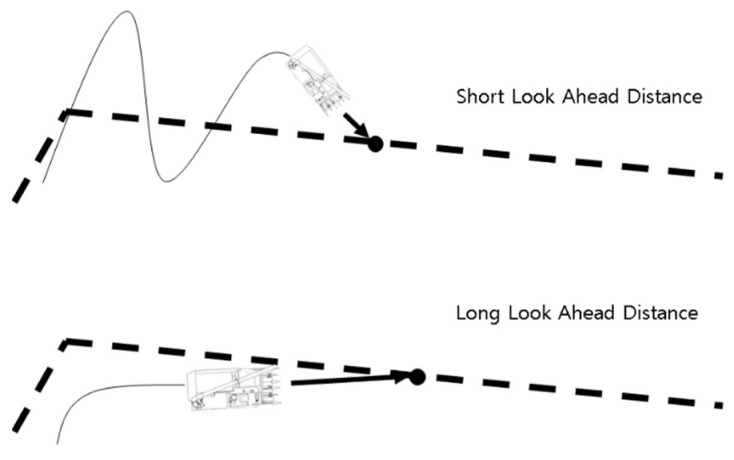
Comparison of driving paths according to Look-Ahead Distance.

On the other hand, if the LAD is large, the performance of following the path decreases but the driving stability increases due to the small steering angle change. Therefore, determining an appropriate LAD for a vehicle is one of the important variables in path tracking. Figure 9 roughly represents the path trackability according to the LAD.

**Figure 10:**
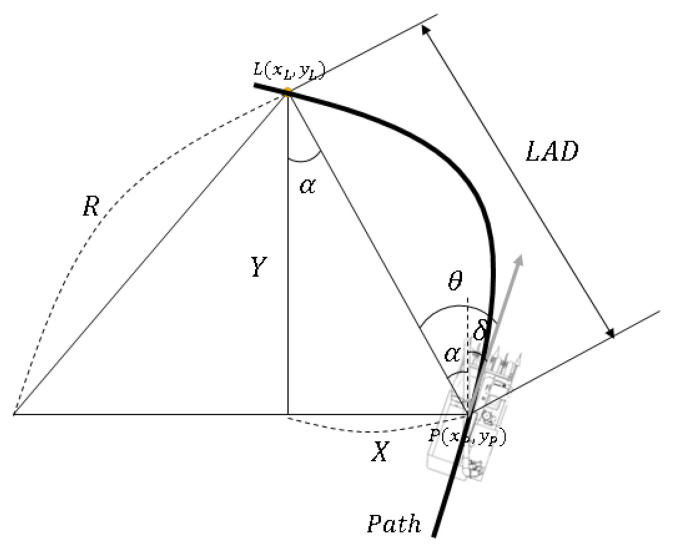
Pure pursuit geometric model for combine harvester.

The existing pure pursuit algorithm calculates the steering angle by drawing an arc using the coordinates of the rear wheel and the target point based on the Bicycle Model. However, in the case of a combine harvester, since the limit of the steering angle and the wheelbase are meaningless, modification is necessary. Considering the characteristics of the combine and using the current coordinate point and the target point, we can model it as shown in Figure 10. The equation for calculating the steering angle through this is as follows:

(14) $$tan\alpha =\frac{X}{Y}$$

(15) $$\alpha =tan^{-1}\left(\frac{X}{Y}\right)$$

(16) θ=−δ−α

X: The *x*-axis distance between the target point and the current coordinates of the combine harvester.

Y: The *y*-axis distance between the target point and the current coordinates of the combine harvester.

θ: The required steering angle of the combine harvester.

δ: The current angle of the combine harvester.

**Figure 11:**
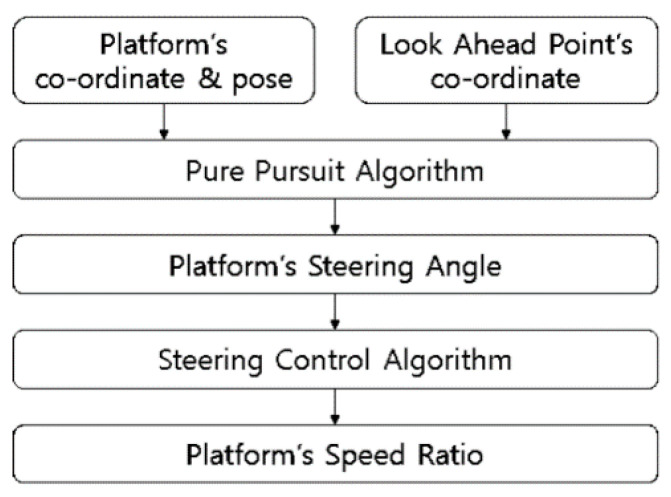
Steering command output flowchart.

The steering control value of the combine harvester is 0 to 2000, and the value of 0 to 999 is the left steering value and means 0.1 to 100% of the left steering output. The value of 1000 means straight driving without separate steering control. Values from 1001 to 2000 are right steering values and mean 0.1 to 100% of right steering output. However, since it is impossible to know the rotational angular speed of the combine harvester according to the value, the experiment was conducted while changing the value from 0 to 2000 in units of 100. As a result of the experiment, the combine harvester turned similarly when the required steering angle was multiplied by the gain value of 35 based on 1000. Figure 11 shows the flow of generating steering command output.

#### 2.4.2. Longitudinal Control

**Figure 12:**
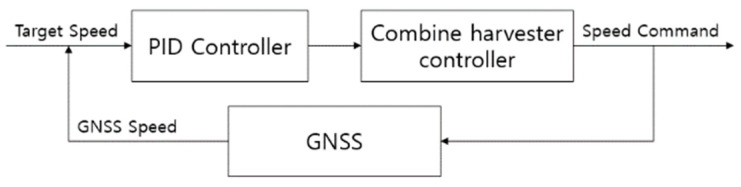
Speed control algorithm using PID Controller.

The longitudinal control of the combine harvester is the speed control, and the speed control is input by dividing the forward output and the backward output. The speed control value is from 0 to 100, and 0 to 100 means a speed output value of 0 to 100%. As the combine harvester harvests rice and its weight increases, it slows down even if it outputs the same speed control value. Therefore, when the target speed is inputted, the speed control value is outputted through the PID control based on the speed obtained from the GPS. Figure 12 shows the PID controller for speed control.

## 3. Results

**Figure 13:**
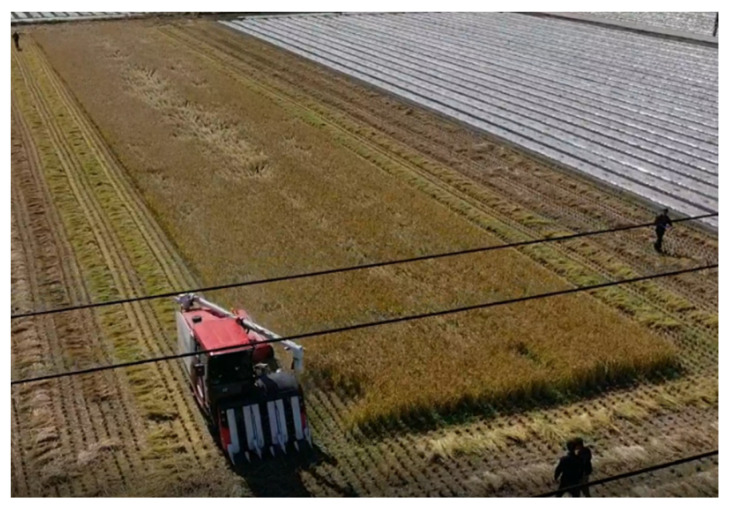
Rice field 730 Boncho-ri, Daeji-myeon, Changnyeong-gun, Gyeongsangnam-do, Korea.

**Figure 14:**
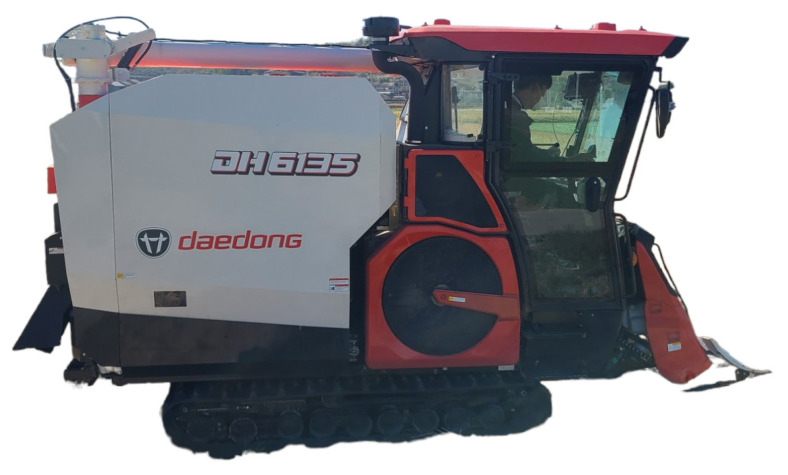
The combine harvester used in the experiment.

The experiment was conducted in an actual rice field by implementing an autonomous driving combine harvester system in a 90 m × 20 m rice field at 730 Boncho-ri, Daeji-myeon, Changnyeong-gun, Gyeongsangnam-do, Korea. Figure 13 shows the rice field where the experiments were conducted. Figure 14 shows the combine harvester that is used in the experiments. In the two experiments, according to the user’s manual, the driving speed was 5~6 km/h on the work path, but it was driven at 1.5 km/h on the turning path.

**Figure 15:**
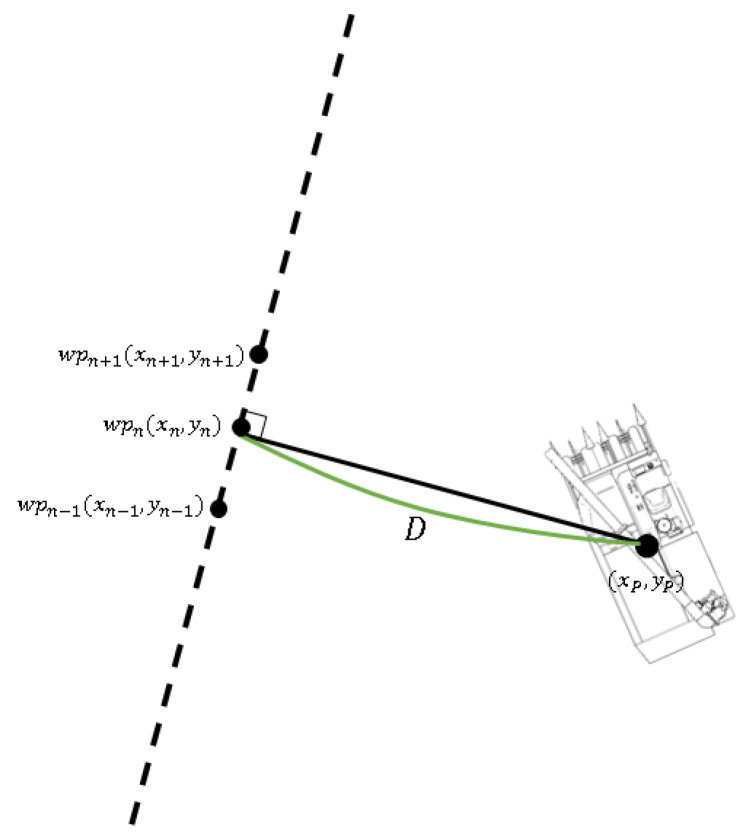
Definition of the path error.

The path error is the difference between the created path and the current position of the combine harvester and is determined by the latitude and longitude received from the GPS. Since the path is composed of point data, it was interpolated in a straight line to obtain the path error accurately. Figure 15 shows the path error, and the formula for obtaining the path error is as follows:

(17) $$\frac{y-y_{n}}{x-x_{n}}=\frac{y_{n+1}-y_{n}}{x_{n+1}-x_{n}}$$

(18) $$y=\frac{y_{n}\left(x-x_{n+1}\right)-y_{n+1}\left(x-x_{n}\right)}{x_{n}-x_{n+1}}$$

(19) $$D=\frac{\left|\left(y_{n+1}-y_{n}\right)x_{p}+\left(x_{n}-x_{n+1}\right)y_{p}+x_{n+1}y_{n}-x_{n}y_{n+1}\right|}{\sqrt{{\left(y_{n+1}-y_{n}\right)}^{2}+{\left(x_{n+1}-x_{n}\right)}^{2}}}$$

### 3.1. Path Tracking without Harvesting Rice

In this experiment, the combine harvester did not perform actual grain harvesting after generating a working path and only performed path tracking.

**Figure 16:**
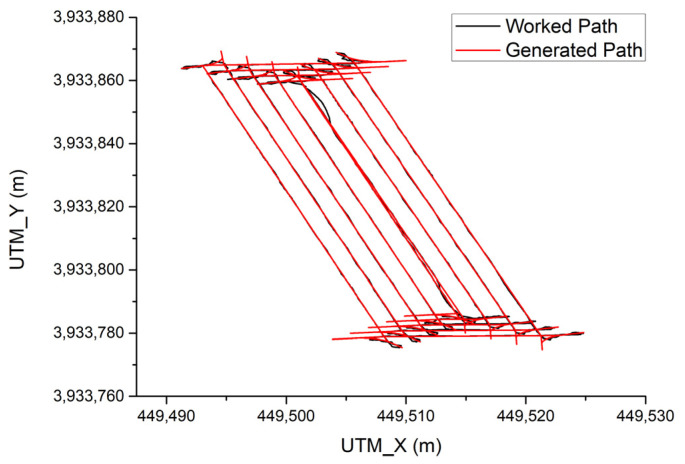
Path tracking result without harvesting rice.

Figure 16 shows the result of the combine harvester only tracking the path without harvesting grain at 5 km/h. The total driving time was 16 min and 40 s, and the total driving distance was 978.6 m. The overall average path tracking error was 0.117 m. The average path tracking error during driving on the working path was 0.052 m, and the average path tracking error during turning driving was 0.207 m.

### 3.2. Path Tracking with Harvesting Rice

In this experiment, the combine harvester harvested grain while driving after generating a working path.

**Figure 17:**
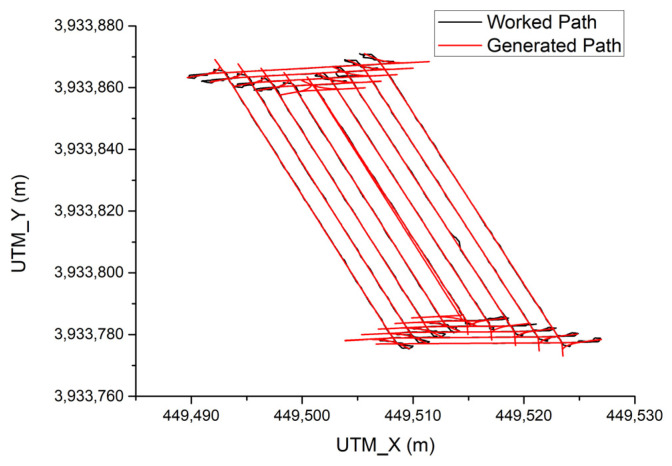
Path tracking result with harvesting rice.

Figure 17 shows the result of the combine harvester tracking the path with harvesting grain at 6 km/h. The total driving time was 16 min and 38 s, and the total driving distance was 1150 m. The driving working width of the combine harvester was 1.8 m, and the total working area was 2070 m^2^. The overall average path tracking error was 0.107 m. The average path tracking error during driving on the working path was 0.038 m, and the average path tracking error during turning driving was 0.195 m. Efficiency was calculated by comparing harvesting results with autonomous driving and the harvesting time and unharvested areas performed by humans. As a result of the calculation, the work efficiency was 76.7%, and there was no unharvested area for both tasks, but it took 1.3 times longer than manual work when working with autonomous driving. This is due to how, in the case of manual work, the driver does not reduce the speed of the combine harvester when turning. However, in the case of autonomous driving, it is driven at 1.5 km/h in consideration of safety because no person is on board.

## 4. Discussion

In this paper, an autonomous driving system for the combine harvester was designed. A path generation algorithm and a path tracking algorithm were developed considering the structural and dynamic characteristics of the combine harvester and the characteristics of the discussion in Korea. As a result of the average error value of the path by executing the actual autonomous driving and harvesting work, an error of about 5 cm occurred during the work, and an error of about 20 cm occurred during the turning. Since the distance between rows of rice is 30 cm, even if an error of about 5 cm occurs during driving, it is judged that it is within the allowable error because there is no non-working area. The results tested in the previous study showed that the average working path error was 0.03–0.04 m at a maximum speed of 3.6 km/h [17] and 0.07 m at a maximum speed of 2.3 km/h [19]. In this paper, the average work path error was 0.04 m, so there was no significant difference, but the work travel speed was 6 km/h and had a stable performance at a higher speed.

Since this research was conducted on the premise of mass production, it had to meet the conditions required by the industry, unlike previous research on autonomous agricultural machines. Since it is impossible to use expensive and precise sensors such as LiDAR and GPS, two GPS sensors are used simultaneously with Moving Baseline RTK and VRS RTK to obtain precision similar to that of expensive sensors. In the case of path generation, since it is created based on the manually driven trajectory, it is greatly influenced by the GPS installed in the combine harvester.

However, in the case of an autonomous driving combine harvester, harvesting work is done on behalf of the worker, so the worker does not have to operate the combine harvester while constantly watching the rows to work. Therefore, it is possible to reduce workers’ fatigue and labor. Since only GPS was used in this study, the performance of the GPS is significant. However, it will be more effective if it is possible to recognize unharvested areas through cameras in the future.

### 4.1. Path Generation Algorithm

Korean rice paddies also transfer the rice from the headland, so harvesting work is required. However, due to the rice paddy bank, the headland has many difficulties generating and tracking paths to work with autonomous driving. When the worker entered the coordinates of the vertex of the rice paddy to perform the harvesting work in the combine harvester, the algorithm generated the global path based on the α-turn. Currently, a person must manually work up to nine rows, the minimum radius of rotation for the α-turn of the combine harvester. Future research on autonomous driving in headland will be conducted to develop a path generation and tracking algorithm capable of fully autonomous driving.

All turns are generated by α-turns only. Therefore, as the remaining work area becomes smaller while continuing to perform the work, the shorter the work driving side, the higher the ratio of distance and time taken for turning driving compared to the work driving, reducing the work efficiency. In order to compensate for these shortcomings, we plan to develop an algorithm that can increase work efficiency by generating paths using other turning methods, such as U-turns, rather than α-turns after a specific area of work when creating paths in the future.

A global route is generated without considering the path for discharging harvested crops. As such, when the combine harvester’s grain tank was full, the worker in the combine harvester manually operated it to discharge the grain. In the future, we plan to develop an algorithm that generates a global path, including the discharge path based on the amount of the grain tank of the combine harvester, as well as the expected yield per distance of the combine harvester and regeneration of the path in real-time for discharge if it is full before the generated discharge path.

### 4.2. Path Tracking Algorithm

The relationship with the ground generated by the combine harvester driving was not considered when developing the path tracking algorithm. However, due to the characteristic of the rice paddy as irrigated farming, the soil has much moisture, so there is ground deformation when the combine harvester is driving, especially when turning. In the future, we plan to develop an algorithm to compensate for errors caused by ground deformation.

Since the combine harvester uses a hydraulic transmission to control the track’s speed, it is not easy to control it precisely. As a result, when tracking a straight driving path, the speed of two tracks moves at a similar speed, so the path tracking error was small because the brake was not used. However, when tracking the turning path, the brake was used to reduce the speed of one track, so the path tracking error was greater than that of tracking the straight path. In the future, we plan to develop speed and steering algorithms that can calibrate for precise path tracking by considering the characteristics of brakes using hydraulic transmissions.

### 4.3. Autonomous Driving System

If GPS data are not received, the combine harvester can not conduct autonomous driving. Due to how the combine harvester uses navigation driving that relies only on GPS for autonomous driving, it is necessary to supplement it. In the next step, we need to develop an algorithm that can establish autonomous driving by distinguishing the work area and non-work area using a camera, so that autonomous driving can be implemented using a camera when the GPS signal is unstable.

Since only GPS sensors were used, no separate sensor can recognize the surrounding environment. Therefore, even if an obstacle or person exists near the combine harvester’s working path, it is impossible to stop or avoid it after recognizing it. In the future, we plan to develop an algorithm that can recognize the environment around the combine and stop it using LiDAR, a camera, or an ultrasonic sensor that can recognize obstacles or people.

## 5. Conclusions

In this paper, the validity of the autonomous driving combine harvester was verified through actual harvest experiments by designing a system of autonomous driving and developing a work path generation and path tracking algorithm considering the characteristics of rice paddies in Korea. In the harvest experiment, it was confirmed that the average path error was within 0.05 m in the working path and within 0.2 m in the turning path, which was available for actual work. However, since the turning speed is lower than the operating speed, it is 76.7% of the efficiency of manual work, so research that can increase the speed of the turning path is needed. In addition, based on the size and shape of the rice paddy, research is needed on the generation of the optimal work path that generates a work path using various turning methods in addition to the α-turn and even the discharge work path.

In the future, based on the algorithms in this paper, we plan to develop a complete path generation and path following algorithm for the combine by supplementing the simplified parts that were not considered in the existing algorithms. In addition, research will be conducted to increase work safety by adding cognitive sensors.

## Figures and Tables

**Table 1:** A specification table of GPS.

Horizontal pos. accuracy	0.01 m + 1 ppm CEP	
Nav. update rate	5 Hz	
Heading accuracy	0.4 deg	
Velocity accuracy	0.05 m/s	
Accuracy of time pulse signal	RMS	30 ns
	99%	60 ns

**Table 2:** RDDF for autonomous combine harvester.

Index	Latitude	Longitude	Velocity	Work Path	Implement
0	451,479.291	3,943,390.395	5	1	1
1	451,479.463	3,943,390.497	5	1	1
2	451,479.634	3,943,390.599	5	1	1
3	451,479.806	3,943,390.700	5	1	1

**Table 3:** Parameters required to create a path.

Field Parameter	Combine Parameters	Path Parameters
Four Field Vertex Points	Implements Width	Interval between Waypoints
	Harvest Operation Speed	Look Ahead Distance
	Turning Operation Speed	Over-run Distance
	Distance between Implement and Control Point	Turning Radius

## Data Availability

Not applicable.
